# Genome Sequence of *Klebsiella pneumoniae* Ecl8, a Reference Strain for Targeted Genetic Manipulation

**DOI:** 10.1128/genomeA.00027-12

**Published:** 2013-01-31

**Authors:** Maria Fookes, Jing Yu, Shyamasree De Majumdar, Nicholas Thomson, Thamarai Schneiders

**Affiliations:** a Wellcome Trust, Sanger Institute, Wellcome Trust Genome Campus, Hinxton, Cambridge, United Kingdom; b Center for Infection and Immunity, Queen’s University Belfast, Belfast, United Kingdom

## Abstract

We report the genome sequence of *Klebsiella pneumoniae* subsp. *pneumoniae* Ecl8, a spontaneous streptomycin-resistant mutant of strain ECL4, derived from NCIB 418. *K. pneumoniae* Ecl8 has been shown to be genetically tractable for targeted gene deletion strategies and so provides a platform for in-depth analyses of this species.

## GENOME ANNOUNCEMENT

*Klebsiella pneumoniae* is an important nosocomial pathogen and ubiquitous colonizer of the environment (1, 2). Given the increasing antimicrobial resistance within this species (3), it is critical to establish phenotypic roles for gene loci linked both directly and indirectly to reduced antimicrobial susceptibility. *K. pneumoniae* strain Ecl8 is of human origin (4) and is amenable to targeted gene deletion (5, 6) using a modified strategy described by Merlin et al. (7). To ensure the accurate mapping of targeted gene deletions using *K. pneumoniae* Ecl8, whole-genome sequencing was undertaken. Our studies indicate that genetic deletions or mutations generated in Ecl8 are widely applicable to other *Klebsiella* strains derived from clinical, environmental, and veterinary niches.

Total bacterial DNA was extracted using the Purelink genomic DNA extraction kit (Life Technologies, United Kingdom), with which the whole genome sequence was determined using both the Illumina HiSeq 2000 and the Roche 454 GS FLX sequencers. The 454 data were produced from an 8-kb-insert library, generating 590,000 reads with an average length of 539 bp by using Ti^+^ chemistry. The 1.2-Gb Illumina data were 100-bp paired reads. A merged assembly was generated by the Newbler program (v 2.6) and improved using IMAGE (8) and iCORN (9). Chromosomal and plasmid sequences were ordered against the genome sequences of *K. pneumoniae* KCTC 2242 (accession no. CP002910 and CP002911), as the KCTC 2242 and Ecl8 chromosomes were found to differ in only 258 single-nucleotide polymorphisms (SNPs), based on a reconstructed phylogenetic tree (10).

The complete genome assembly for *K. pneumoniae* Ecl8 is composed of a chromosome of 5,324,709 bp in 5 contigs, including the 454 scaffolds and the 206,102-bp-long plasmid in a single scaffold (3 gaps). The *N*_50_ values for the chromosome and plasmid were 279,996 and 52,921 bp, respectively, and the largest contig values were 449,727 and 77,767 bp. Published genome sequences of *K. pneumoniae* strains 342 and KCTC 2242 were used to inform annotation, prior to manual curation.

The chromosome of *K. pneumoniae* Ecl8 has a G+C content of 57.2%. Of its 5,006 predicted coding sequences (CDS), 44 are pseudogenes sustaining mutations likely to ablate function; 85 tRNAs, 25 rRNAs, and 88 sRNAs are also predicted. TBLASTx comparisons between the KCTC 2242 and Ecl8 chromosomes showed that the latter carries two insertions: the first (8.6 kb) carries the beta-lactamase resistance gene *ampH* and the ATP-binding cassette (ABC) transporter domain-containing gene *smbA*, among others; the second insertion is a 39.2-kb novel prophage inserted alongside tRNA^Arg^. Database analysis showed that this prophage is similar in regions to the *Salmonella enterica* serovar Typhimurium ST64B prophage, particularly in relation to capsid-encoding genes; there are no obvious regions of low G+C content within this prophage or gene or protein motifs that indicate the carriage of cargo genes associated with virulence or lipopolysaccharide modification. The plasmid is identical to the previously reported *K. pneumoniae* plasmid pKTCT2242 except for two additional insertion sequence elements.

### Nucleotide sequence accession numbers. 

Raw data were submitted to the European Nucleotide Archive (ENA) with sample accession no. ERS088743. Genome assemblies, qualifying as an improved high-quality draft standard (11) of the chromosome and plasmid of *Klebsiella pneumoniae* Ecl8, have EMBL accession numbers HF536482 and HF536483, respectively.
